# High-throughput dual-colour precision imaging for brain-wide connectome with cytoarchitectonic landmarks at the cellular level

**DOI:** 10.1038/ncomms12142

**Published:** 2016-07-04

**Authors:** Hui Gong, Dongli Xu, Jing Yuan, Xiangning Li, Congdi Guo, Jie Peng, Yuxin Li, Lindsay A. Schwarz, Anan Li, Bihe Hu, Benyi Xiong, Qingtao Sun, Yalun Zhang, Jiepeng Liu, Qiuyuan Zhong, Tonghui Xu, Shaoqun Zeng, Qingming Luo

**Affiliations:** 1 Britton Chance Center for Biomedical Photonics, Wuhan National Laboratory for Optoelectronics-Huazhong University of Science and Technology, 1037 Luoyu Road, Wuhan 430074, China; 2 Department of Biomedical Engineering, Key Laboratory for Biomedical Photonics of Ministry of Education, Huazhong University of Science and Technology, 1037 Luoyu Road, Wuhan 430074, China; 3 Howard Hughes Medical Institute and Department of Biology, Stanford University, 385 Serra Mall, Stanford, California 94305, USA

## Abstract

The precise annotation and accurate identification of neural structures are prerequisites for studying mammalian brain function. The orientation of neurons and neural circuits is usually determined by mapping brain images to coarse axial-sampling planar reference atlases. However, individual differences at the cellular level likely lead to position errors and an inability to orient neural projections at single-cell resolution. Here, we present a high-throughput precision imaging method that can acquire a co-localized brain-wide data set of both fluorescent-labelled neurons and counterstained cell bodies at a voxel size of 0.32 × 0.32 × 2.0 μm in 3 days for a single mouse brain. We acquire mouse whole-brain imaging data sets of multiple types of neurons and projections with anatomical annotation at single-neuron resolution. The results show that the simultaneous acquisition of labelled neural structures and cytoarchitecture reference in the same brain greatly facilitates precise tracing of long-range projections and accurate locating of nuclei.

## Abstract (web summary)

High-throughput imaging methods for brain-wide connectome mapping with precise location reference have been lacking. Here authors report a method that allows simultaneous acquisition of fluorescently labelled neurons and cytoarchitectural landmarks in the same mouse brain at the single-cell resolution.

## Graphical abstract

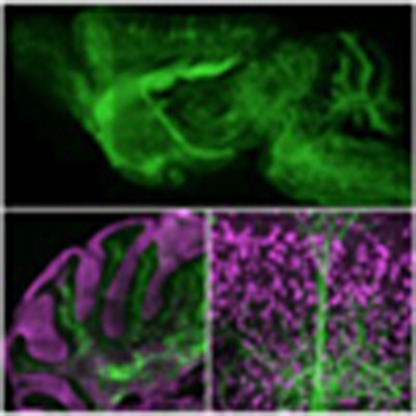

At the cellular level, the connectome precisely annotates a comprehensive map of neural connections in the brain and significantly increases current understanding of how functional brain states emerge from underlying structural substrates. Mapping a cellular mouse connectome requires centimetre-scale imaging with axon resolution. Combined with physical sectioning, Li *et al*.^1^, Ragan *et al*.^2^ and Gong *et al*.^3^ pioneered whole-brain optical microscopic imaging. Neurons with different functions have different sizes, shapes and locations, and even neighbouring neurons of the same cell type differ in their morphologies and projection patterns^4^,^5^. Thus, orienting neural projections at single-cell resolution is required for both the precise annotation and accurate identification of the spatial organization of neural structures. To locate neurons and neural circuits, researchers usually map brain images to coarse axial-sampling planar reference atlases. However, individual differences at the cellular level likely lead to position errors, making the precise annotation and accurate identification of projections at single-cell resolution difficult.

Here, we develop an automatic microscopy method called the brain-wide positioning system (BPS) for dissecting and locating neural structures with cytoarchitectonic landmarks at a single-cell resolution. BPS involves a whole-brain real-time counterstain protocol for simultaneously staining cytoarchitectonic landmarks during imaging and a high-throughput multi-channel brain-wide imaging system, termed wide-field large-volume tomography (WVT), for accelerating imaging acquisition at single-cell resolution. We obtain five full-volume, dual-colour mouse brain data sets of cell-type-specific fluorescent protein-expressing neurons and neuronal projections and subsequently reconstruct the three-dimensional (3D) fine structure of these neurons at the axon level using cellular landmarks. This standardized precision imaging approach facilitates quantitative brain-wide studies of 3D neuronal morphology, dendritic arbor and bouton distributions, axonal projection densities and distances and neuronal cell types, based on cytoarchitectonic landmarks at the cellular level. We propose that this method represents a routine tool for highly accurate and precise analysis of the cellular connectome.

## Results

### Brain-wide positioning system

**Figure 1:**
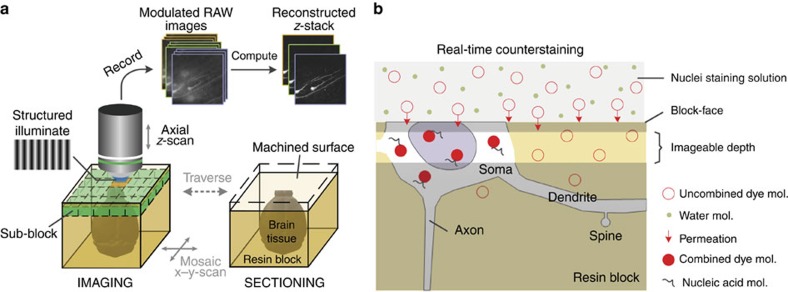
Principle of BPS. (**a**) BPS Data acquisition overview. The resin-embedded sample moves between the SIM and the microtome. The SIM acquires three raw images to reconstruct an optical section and axially scans for Z-stack imaging over a certain range. The entire coronal section is imaged in a mosaic manner. (**b**) Schematic representation of the real-time cytoarchitecture staining. The dye molecules penetrate into the fresh resin-embedded sample surface and immediately combine with the nucleic acids inside the cell body. The soma is then visualized.

As a proof of principle, we revealed the spatial distribution with anatomical annotation of an 8-week-old *Thy1*-GFP M-line transgenic mouse^6^ using BPS (Fig. 1 and Supplementary Fig. 1). The resin-embedded whole-brain sample was then fixed in the anterior-posterior (A-P) direction on a 3D translation stage. The WVT system employed wide-field two-channel fast structured illumination microscopy (SIM) to accelerate imaging acquisition. SIM, which was first demonstrated by Neil *et al*.^7^, has optical sectioning capability comparable to confocal microscopy. The system automatically performed brain-wide data acquisition. The 3D translation stage moved the sample to the microtome, and subsequently the microtome removed the superficial layer, revealing a smooth surface. The sample was then moved to fast SIM, and images were acquired through mosaic scanning in each layer. To avoid the compromised effect of sectioning marks on the machined surface, we set the imaging plane slightly below the surface, generally at 1–2 μm. The sectioning-imaging cycles were repeated until entire brain acquisition was completed (Fig. 1a). To achieve whole-brain counterstaining, we immersed the sample in a fluorescent nuclear staining solution during whole-brain imaging. After sectioning, the dye immediately penetrated the fresh surface, combining with nucleic acid inside cell bodies and staining the soma, proximal dendrites and axon hillocks (Fig. 1b and Supplementary Figs 2, 3). To improve the detection of weak fluorescence signals in thick tissue through SIM, we modified the sample processing protocol. We added a light absorber, Sudan black B (SBB)^8^, to the embedded resin to inhibit background fluorescence and enhance the signal-to-noise ratio of the SIM imaging (Supplementary Fig. 4). We also used chemical reactivation of the quenched fluorescent protein^9^ on the surface of the sample to enhance the in-focus GFP signal.

### Whole-brain neuron-mapping with cytoarchitecture

**Figure 2:**
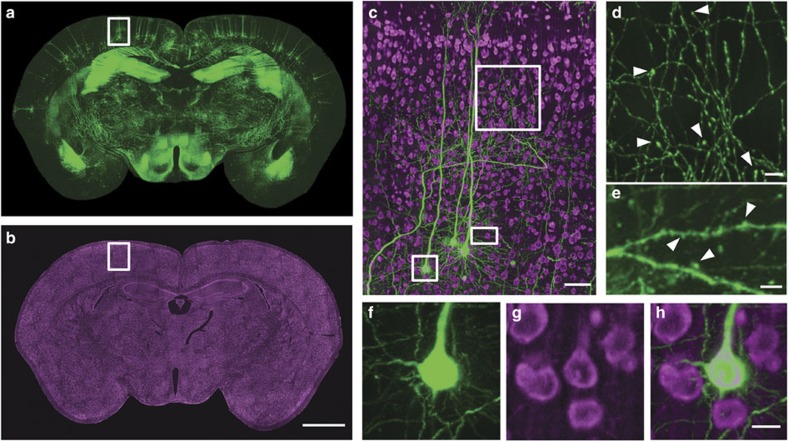
Brain-wide dual-colour imaging of a *Thy*1-GFP M-line mouse brain. (**a**,**b**) Maximum intensity projections of the coronal sections. The projections were 400 (**a**) and 1 μm (**b**) thick. (**c**) Merged images indicated by the white boxes in **a**,**b**. (**d**) An enlarged view of the area indicated by the upper white square in **c**, demonstrating the visualization of axonal boutons, indicated using arrowheads. (**e**) An enlarged view of the area indicated by the white rectangle in **c** demonstrating visualization of dendritic spines, indicated using arrowheads. (**f**,**g**) Raw signals of the enlarged views of the area indicated by the lower white square in **c**. (**h**) Merged image, demonstrating accurate co-localization at single soma resolution using PI staining. (**c**,**h**) The projection thicknesses of GFP and PI were 400 μm and 1 μm, respectively. Scale bars, (**a**,**b**) 1 mm; (**c**) 50 μm; (**d**) 40 μm; (**e**) 5 μm; and (**f**–**h**) 10 μm.

**Figure 3:**
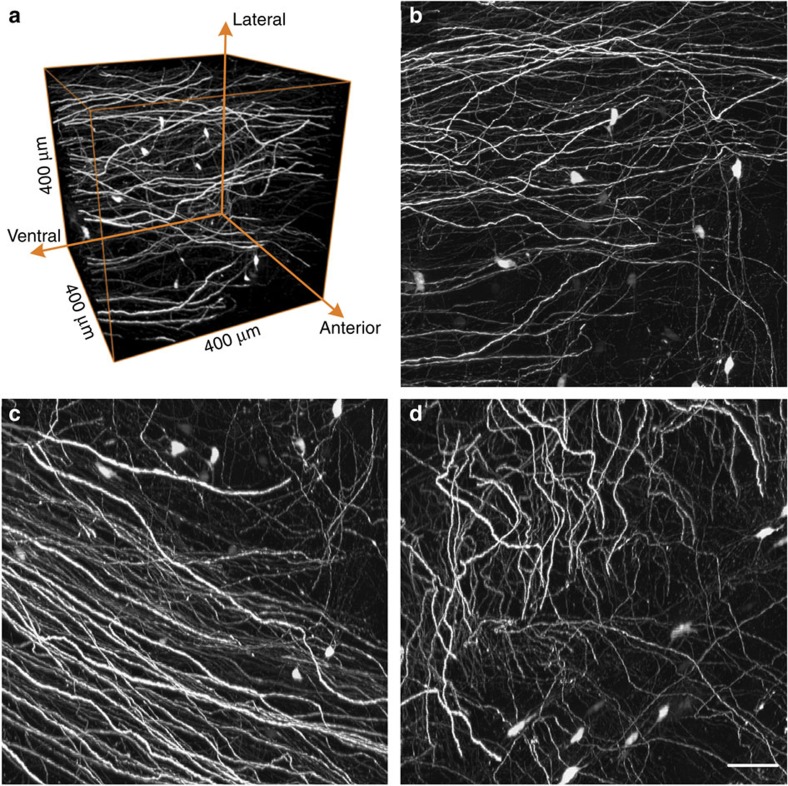
Continuous neural projections with high resolution. (**a**) 3D reconstruction of a GFP-labelled image block without preprocessing. The image block is selected from the same whole-brain data set of Fig. 2 and located in the thalamus. (**b**–**d**) Maximum intensity projections on transverse, sagittal and coronal sections, accordingly. Each projection depth is 400 μm. Scale bar, 50 μm.

The whole-brain data set from a *Thy1*-GFP M-line transgenic mouse, including GFP-labelled neurons and propidium iodide (PI)-stained cytoarchitecture, was acquired at a voxel resolution of 0.32 × 0.32 × 1 μm in a coronal plane. The images of the typical structure of the hippocampus, cortex and cellular layers of the cortex (Fig. 2a,b) demonstrate the method used in the present study to identify classical regions through PI-stained landmarks. Neuron arbors, axon boutons and dendritic spines were distinguished in the reconstruction of neurons (Fig. 2c–e). The overlapping of soma with GFP-labelled neurons and PI-stained cellular nuclei (Fig. 2f–h) illustrated single-cell co-location accuracy in the same brain using this BPS method. The sagittal reconstruction of continuous projections demonstrated that the high resolution and self-registration of BPS guarantee the data integrity of the whole-brain data set (Supplementary Fig. 5). Precision imaging through BPS not only reveals the complexity of fibre orientations, even in a small tissue volume (400 × 400 × 400 μm), but also distinguishes individual axons in the dense fibre bundle (Fig. 3 and Supplementary Movie 1).

### Reconstruction and localization of single pyramidal neurons

**Figure 4:**
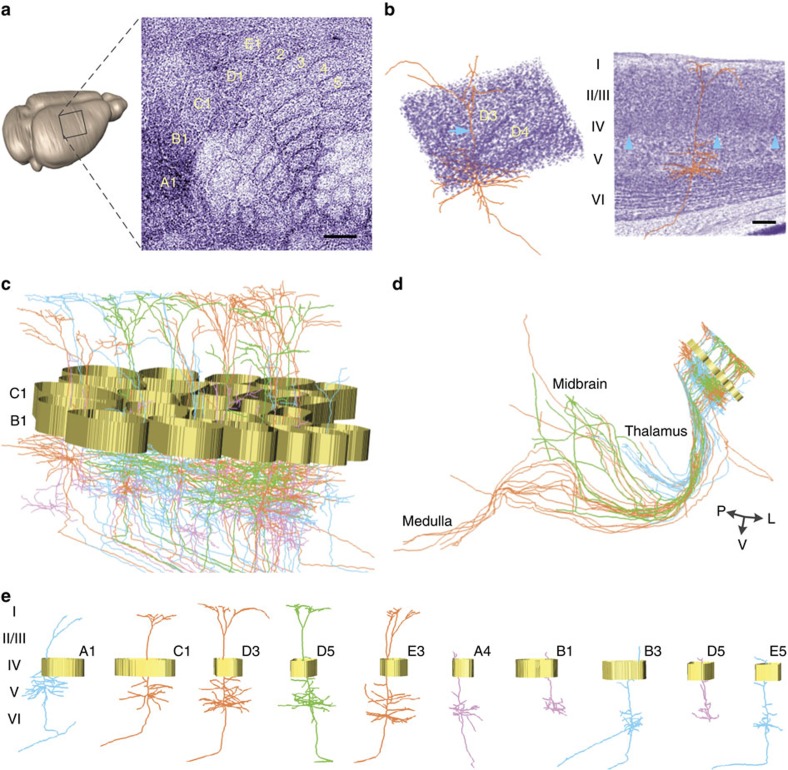
Localization and reconstruction of pyramidal neurons in the *Thy*1-GFP M-line mouse barrel field. (**a**) PI-channel volume rendering and the 220-μm-thick perspective projection of the barrel field of the right hemisphere, showing layer IV cytoarchitecture. Scale bar, 200 μm. (**b**) A reconstructed pyramidal neuron and associated cytoarchitecture reference. (left) Neuron and the local layer IV cytoarchitecture of the D3 and D4 columns (410 × 340 × 80 μm image stack). The apical dendrites of this neuron are located in the barrel hollow of D3. The blue arrow indicates the location where the neuron passes through the upper boundary of the image stack. (right) The cytoarchitecture from layer I to the corpus callosum surrounding the same neuron as shown on the left. The blue arrowheads indicate barrel walls. Image stack volume size: 760 × 850 × 150 μm. Scale bar, 100 μm. (**c**) 3D reconstruction of the barrel walls and 50 representative neurons located in the barrel field. The barrel walls were reconstructed according to PI-stained images and represented as gold loops (height: 80 μm). (**d**) The distribution of all long-range projection neurons among the 50 representative neurons. The thalamus, midbrain and medulla are the major projection regions. L, left; P, posterior; and V, ventral. (**e**) Ten reconstructed neurons (**c**) and corresponding barrel columns. The five neurons shown on the left are long-tufted pyramidal neurons, and the remaining five neurons are short-sparse neurons. The orange neuron located in D3 is the same neuron shown in **b**. Blue, green and orange colours represent neurons with axons extending the furthest to the striatum or thalamus, the midbrain and the pons or medulla, respectively. Pink denotes local projection neurons.

Furthermore, to illustrate the accurate co-localization of the same neuron types with cellular landmarks in the same region, we examined classical function columns and determined the complete 3D morphology of barrel cortex layer V/VI pyramidal neurons. The cellular organization of each cortical barrel column is whisker-specific^10^. The function, intrinsic connectivity and cellular composition of the barrel column were characterized in detail. We acquired another dual-colour whole-brain data set from *Thy1*-GFP M-line transgenic mouse at a voxel size of 0.32 × 0.32 × 2 μm in 77 h (Supplementary Movie 2). The raw data set was larger than 10.9 terabytes, including 4,834 coronal sections for each channel. We clearly identified regularly arranged ‘barreloids' in layer IV of the primary somatosensory cortex (S1) using perspective projections of the PI-channel image stack (Fig. 4a). The image of the PI-stained cortex illustrates a typical six-layer structure (Fig. 4b). The reconstruction of a GFP-expressing projection neuron located in layer V of the barrel field of S1 shows the typical morphology of cortical pyramidal neurons (Fig. 4b and Supplementary Movie 3). The apical dendrites ascend through the barrel in layer IV, generating the tuft observed in layer I. Moreover, we identified and produced 3D reconstructions of all barrel columns in the mouse barrel cortex according to the PI signal and then located the reconstructed 50 layer V/VI pyramidal neurons in the barrel field (Fig. 4c). The results showed various morphological distributions of these neurons among different barrels. The ability of BPS to continuously obtain high-quality images facilitates the tracing of individual pyramidal neurons (Supplementary Movies 4 and 5). We classified the reconstructed neurons as local (*n*=14) and long-range (*n*=36) projection neurons according to the extent of the axonal arborizations. For long-range projection neurons, the axons extended toward the striatum or thalamus (*n*=16), midbrain (*n*=6) and pons or medulla (*n*=14), and most of these neurons had other collaterals that projected to distinct brain regions (Fig. 4d). In addition, we classified these 50 pyramidal neurons as long-tufted (*n*=24) and short-sparse (*n*=26) neurons (Fig. 4e). The apical dendrites of the long-tufted neurons ascended to layer I and had abundant arbors, whereas those of the short-sparse neurons did not reach layer I and exhibited only rare branches. All local projection neurons were short-sparse neurons. Details on the localization and 3D reconstruction of these 50 neurons throughout the entire brain are shown in Supplementary Movie 6. These results showed that the spatial relationships between pyramidal neurons and barrel columns in the mouse barrel cortex are diverse, indicating that these individual neurons might play different roles in whisker movement. Compared with traditional single-cell tracing, BPS method has two advantages. The morphology reconstructions of neurons are consecutive and complete, including local and long-range axons. In addition, simultaneously imaging neural structure and cytoarchitecture during whole-brain imaging enables to locate the reconstructed structures according to their own cytoarchitecture.

### Quantitative morphology analysis of pyramidal neurons

**Figure 5:**
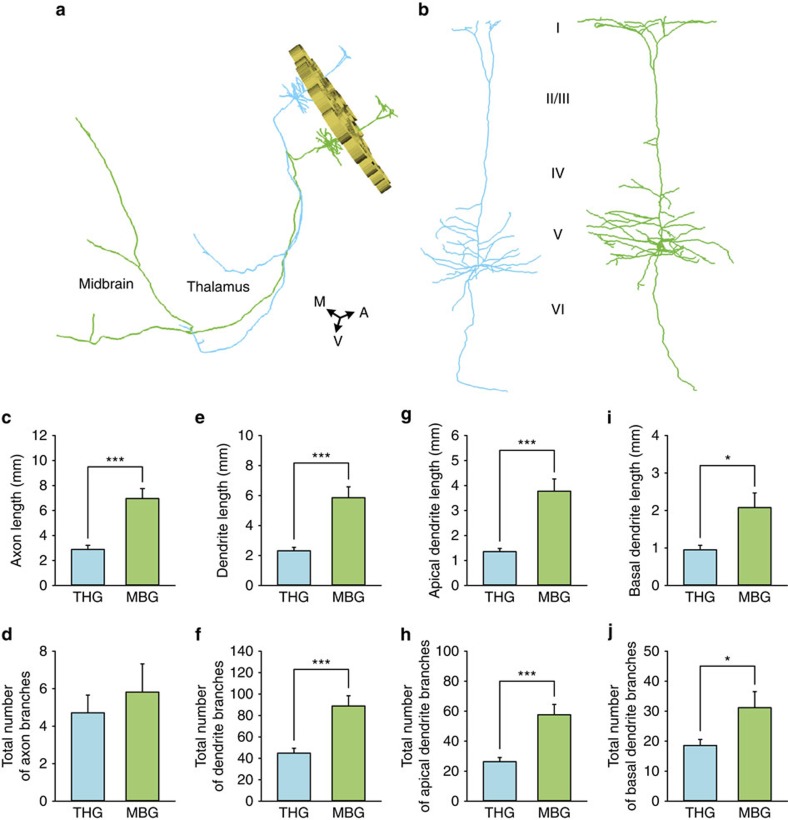
Quantitative comparison of the fine morphology of neurons. (**a**) 3D reconstruction of the barrel walls and two representative long-range projection neurons located in the barrel cortex. The barrel walls were reconstructed according to PI-stained images and represented as gold loops (height: 80 μm). The blue neuron was localized to the D5 barrel column and projected to the thalamus. The green neuron was localized in the E2 barrel column and projected to the midbrain. A, anterior; M, middle; and V, ventral. (**b**) The enlarged views of the dendrites of the two neurons in (**a**) showing the distinct dendrite complexities of the projection neurons in the two groups. (**c**,**d**) Histograms of the axonal lengths (*P*<0.001) and branch numbers (*P*=0.520) of the long-range neurons, respectively. THG (*n*=15) and MBG (*n*=11) represent the neurons projecting to the thalamus and midbrain, respectively. (**e**,**f**) Histograms of the dendrite lengths (*P*<0.001) and branch numbers (*P*<0.001) of long-range neurons, respectively. (**g**,**h**) Histograms of the apical dendrite lengths (*P*<0.001) and branch numbers (*P*<0.001) of long-range neurons, respectively. (**i**,**j**) Histograms of the basal dendrite lengths (*P*=0.017) and branch numbers (*P*=0.045) of long-range neurons, respectively. We performed Student's *t*-test on two groups by SPSS 22. Error bars were defined as s.e.m. Confidence level was set to 0.05 (*P* value). * represents *P*<0.05, and *** represents *P*<0.001.

We also orientated and traced long-range projection neurons in the barrel cortex in the first *Thy*-1 GFP data set. Consistent with the results from the second mouse brain, the axons of long-range projection neurons primarily extended toward the striatum or thalamus (*n*=5), midbrain (*n*=5), and pons or medulla (*n*=3). Using these two data sets, we quantitatively compared the fine morphology of neurons projecting to the thalamus (thalamus group (THG), *n*=21) with those projecting to the midbrain (midbrain group (MBG), *n*=11) (Fig. 5a and Supplementary Table 1). The axonal lengths of the MBG were more than double those of the THG (Fig. 5c), whereas the numbers of axonal branches were similar between the two groups (Fig. 5d). The dendritic complexity of the neurons was significantly different between the two groups. Both the dendrite lengths (Fig. 5e) and branch numbers (Fig. 5f) of the neurons in the MBG were much larger than those in the THG. Furthermore, we analysed and compared the morphology of the apical and basal dendrites in the two groups. The two groups had obvious differences in both apical dendrite lengths (Fig. 5g) and branch numbers (Fig. 5h). For the basal dendrites, the lengths of the neurons in the two groups were obviously different (Fig. 5i), although there was no obvious difference in the branch numbers between the two groups (Fig. 5j). These results showed that the neurons projecting to the midbrain have longer axons and more complicated dendrite structures than the neurons projecting to the thalamus (Fig. 5a,b). Furthermore, these results indicate that in the barrel cortex, pyramidal neurons with longer axonal projections have a more complicated dendritic structure.

### Mapping long-range projections with nuclei locations

**Figure 6:**
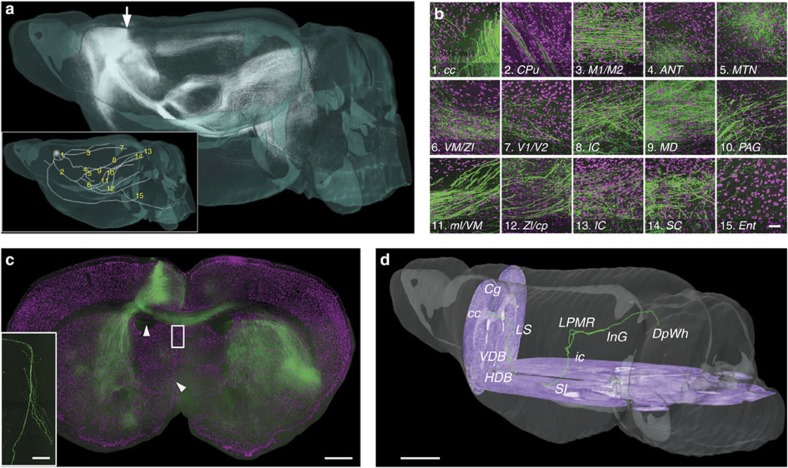
Anterograde tracing and localization of brain-wide neural projections. (**a**) Anterograde projections of AAV-GFP-labelled Cg neurons in the entire brain. The inset shows the main pattern of the Cg projecting to the whole brain, and the 15 passing regions are indicated in the global observation at low resolution. The grey sphere indicates the injection site. (**b**) Merged local maximum intensity projections of the sagittal planes of the corresponding positions indicated in **a**. Passing regions: (b1) corpus callosum, (b2) caudate putamen, (b3) primary/secondary motor cortex, (b4) anteroventral thalamic nucleus and anterodorsal thalamic nucleus, (b5) anteromedial thalamic nucleus and paratenial thalamic nucleus, (b6) ventromedial thalamic nucleus, zona incerta dorsal part (ZID), zona incerta ventral part (ZIV), and reticular thalamic nucleus, (b7) primary/secondary visual cortex, (b8) inferior colliculus (ic), (b9) mediodorsal thalamic nucleus central part, mediodorsal thalamic nucleus dorsal part and mediodorsal thalamic nucleus lateral part, (b10) periaqueductal gray, (b11) medial lemniscus and ZID, (b12) ZID/ZIV and cerebral peduncle, (b13) ic, (b14) superior colliculus and (b15) entorhinal cortex. The size of each panel is 300 × 320 μm. Green represents the maximum intensity projections of GFP-labelled axons. The projection thickness shown in images 3, 5, 6, 9, 13 and 14 is 32 μm, and the projection thickness of the other images is 64 μm. Magenta represents PI-stained cells (2 μm thickness). (**c**) Projection pattern from Cg neurons on the coronal plane indicated with an arrow in (**a**). Green represents the maximum intensity projection of GFP-labelled projections (960 μm total thickness). Magenta represents PI-stained cells (2 μm thickness). The inset is an amplified image of the block shown in (**c**) containing sparse axons. (**d**) The reconstructed and localized axon indicated with arrowheads in (**c**). The cell body is located in the Cg region, and the axon connects to DpWh. Additionally, the axon bifurcates in the ic and LPMR brain regions. Purple represents the PI-counterstained cytoarchitecture background. Scale bar, 50 μm (**b**); 500 μm (**c**); 50 μm (the inset in **c**); and 100 μm (**d**).

The co-localization of axonal long-range projections with cellular landmarks is a prerequisite for the precise analysis of the cellular connectome. To illustrate the accurate identification of the nuclei that the axons pass through and precise tracing of single axons, we performed two-channel whole-brain imaging at a voxel size of 0.32 × 0.32 × 2 μm on an 8-week-old C57BL/6J mouse injected with adeno-associated virus vector plasmid (AAV, expressing GFP)^11^ in the cingulate cortex (Cg). We reconstructed GFP-labelled axons and conducted anterograde tracing of the brain-wide projections from the Cg (Fig. 6a and Supplementary Movie 7). We determined the destination nuclei according to the PI signal, and the results highlighted projection connectivities between the Cg region and the accumbens nucleus (Acb), posterior pretectal nucleus (PPT), ventral tegmental area (VTA) and other brain regions and such as the contralateral cortex and subcortical brain areas, consistent with previous studies^12^,^13^. Most notably, the high resolution of BPS revealed the distribution and location of the axon arbors and cells in any brain region. We identified 15 nuclei through which the projections passed (the inset of Fig. 6a). These regions received different projection patterns from the Cg, and the morphology and density of the cells varied among different areas (Fig. 6b). In particular, the BPS method obtained fine and accurate whole-brain imaging at a high spatial resolution (Fig. 6c), thereby facilitating not only the distinction of dense GFP-labelled neurons but also the tracing of single-axon pathways. We extracted a 541 × 667 × 180 μm data cube at the injection site and counted 746 AAV-labelled neurons (Supplementary Fig. 6 and Supplementary Movie 8). We traced an axon from the Cg to the deep white layer of the superior colliculus (DpWh) (the inset in Fig. 6c,d, Supplementary Fig. 7 and Supplementary Movies 9 and 10). This axon projected successively through eight brain regions, including corpus callosum (cc), lateral septal nucleus (LS), the nucleus of the vertical limb of the diagonal band (VDB), the horizontal limb of the diagonal band (HDB), substantia innominata (SI), inferior colliculus (ic), lateral posterior thalamic nucleus, mediorostral part (LPMR) and the intermediate grey layer of the superior colliculus (InG), indicating that the cingulate has direct connections with brain regions involved in vision, pain and attention regulation. These results demonstrated that the BPS method has an advantage in tracing and co-localizing brain-wide dense projections.

Notably, different from previous method for axon tracing^14^, the high-spatial resolution of BPS enables us to perform the microanatomical quantitative analysis of subcellular structure. To determine whether the terminal axon distribution could be revealed through BPS, we analysed the terminal axon arbors and boutons from a long-range projection neuron of the hypothalamus in the cortex (Fig. 2f). In a 128 × 128 × 400 μm cube of layer II/III, the length of the axon arbor is 3,221.6 μm, and the number of boutons is 121. These quantitative results demonstrate that BPS not only facilitates the determination of connections among regions but also evaluates connection efficiencies.

**Figure 7:**
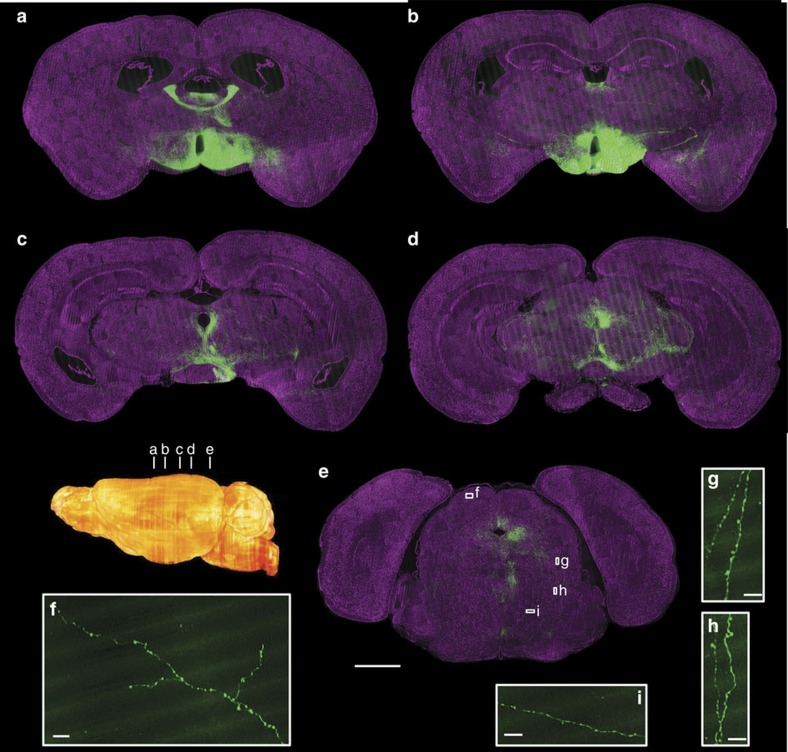
Whole-brain imaging of GFP-labelled neurons in a *SOM Cre*-line mouse brain. (**a**–**e**) Maximum intensity projections of different coronal sections showing the projection patterns with cytoarchitectonic references. The inset shows locations of images shown in **a**–**e**. The projection thicknesses of GFP-signal were 60 μm (**a**,**b**), 120 μm (**c**) 200 μm (**d**) and 100 μm (**e**). Magenta represents PI-stained cells (2 μm thickness). (**f**–**i**) Representative raw images showing the fine structure, such as axon boutons, of the long-range projections shown in (**e**). Scale bar, 1 mm (**a**–**e**) and 15 μm (**f**–**i**).

In addition, we injected 90 nl of AAV-FLEX (expressing GFP)^15^ into the posterior region of the anterior hypothalamic area of a *SOM*-*Cre* (ref. 16) mouse and imaged the entire brain using dual-colour channels with BPS at a voxel size of 0.32 × 0.32 × 2 μm. The results showed the whole-brain projection patterns of the specific neuron type in the injected brain area (Fig. 7) and suggested that BPS in combination with different labelling methods facilitates the analysis of the locations and functions of specific neurons and neural circuits.

**Figure 8:**
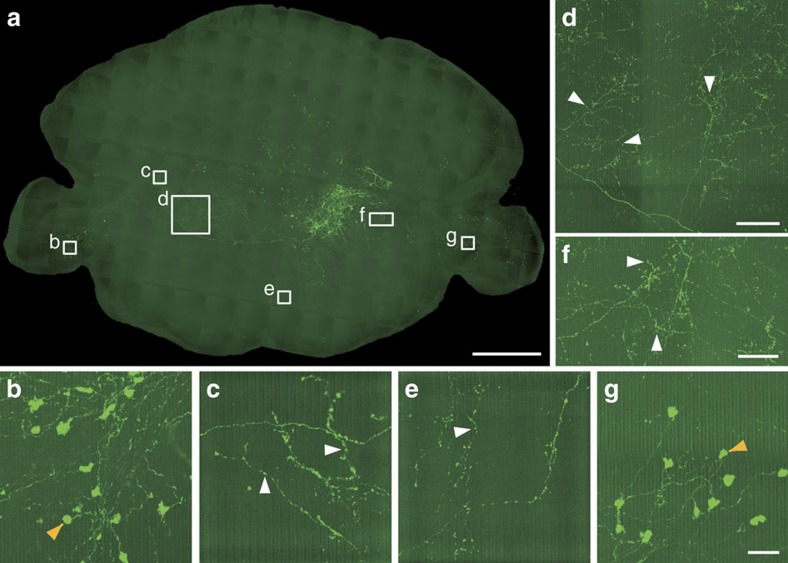
Reconstruction of sparse-labelled brainstem neurons with long-range projections. (**a**) Maximum intensity projections of the coronal sections. The projection thickness was 900 μm. (**b**–**g**) Enlarged views of the single axon arbor and axon terminals of the neurons shown in **a**. The reconstructed boutons are indicated as white arrowheads. The large axon terminals were indicated as orange arrowheads. Scale bar, 1 mm (**a**); 100 μm (**d**); 50 μm (**f**); and 25 μm (**b**,**c**,**e**,**g**).

Moreover, we labelled brainstem neurons with long-range projections to olfactory bulb of a 7-week-old wild-type mouse. We imaged the entire brain using dual-colour channels through BPS at a voxel size of 0.32 × 0.32 × 2 μm (Fig. 8). Sparse labelling in combination with BPS facilitates the visualization and orientation of local and long-range projections of brainstem neurons with cytoarchitecture in the whole brain (Fig. 8a and Supplementary Fig. 8). We identified the axon terminals of several neurons (Fig. 8b–g). Thus, these results explicitly show that these long-range neurons project to the bilateral paraflocculus (PFI) as well, and possess large axon terminals (Fig. 8b,g). The labelled neurons were so sparse that the detailed branches and axon terminals from a single axon could be identified (Fig. 8d).

## Discussion

Here, we report a high-throughput method for the precise reconstruction and accurate localization of specific labelled neurons and projections in the whole brain. We observed the integrated cytoarchitecture of barrel columns in the barrel cortex using real-time counterstaining during whole-brain imaging and traced layer V/VI pyramidal neurons. In addition, we precisely traced a representative single-neuron projection on these PI-counterstained landmarks and identified the nuclei through which this projection passed. The diverse and complex spatial relationships between these neurons and nuclei suggest the necessity of reconstructing and analysing neuronal morphology and neural connections with accurate anatomical annotation. In this regard, BPS provides two major advantages.

First, the acquisition of cytoarchitecture landmarks in whole-brain imaging is simple and feasible. In traditional neurobiological experiments, the registration of neural images to a planar brain reference atlas is typically required. Individual differences among subjects, unavoidable deformation during tissue preparation, and interval-sampling planar reference atlases inevitably generate location errors in these forced registrations. Thus, staining the cytoarchitecture landmarks and labelling the neural structures in the same brain are necessary to avoid these errors. However, low-permeability nucleic acid dyes, such as PI and DAPI, are traditionally used in slide staining to label cytoarchitecture landmarks, and these stains are difficult to use when staining the whole mouse brain. Here, we propose a new concept of real-time counterstaining during whole-brain imaging, rather than whole-brain counterstaining. The low permeability of nucleic acid dyes results in superficial staining during whole-brain imaging without background interference from deep layers in the same channel. Moreover, the sectioning-staining-imaging cycle guarantees consistent PI staining in all coronal planes. This real-time staining approach avoids additional sample preparation. The stained cytoarchitecture and labelled neurons in the same field of view (FOV) are simultaneously imaged. Thus, neither additional data acquisition time for anatomical landmarks nor extra two-channel registration is needed. This concept can be applied to other mechanical section-based whole-brain imaging techniques, such as serial two-photon (STP) tomography and fluorescence micro-optical sectioning tomography (fMOST), for acquiring anatomical references in the same brain.

Second, WVT efficiently shortens the time required for data acquisition during full-volume whole-brain imaging at single-neuron resolution. The time required for data acquisition is one of the decisive factors for the use of whole-brain imaging as a routine approach in neuroanatomical studies. Currently, STP is considered the fastest approach; however, this approach sacrifices the continuity of the whole-brain imaging data set. fMOST and 2p-fMOST (ref. 17) distinguish the detailed neural structures in full-volume whole-brain imaging, requiring more than 1 week for completion. As all these techniques employ a point-scanning approach, it is challenging to improve imaging throughput. Taking advantage of the high throughput of the wide-field and the background inhibition of SIM, BPS achieves full-volume imaging of the mouse brain with high resolution within 3 days. The high continuity and self-registration of the whole-brain imaging data set through BPS facilitates the tracing of the detailed morphology of neurons, including axons, dendritic arbors, boutons and spines in the whole brain. With the rapid development of an objective with a larger FOV, a camera with a larger sensor size, and a 3D stage with longer travel, we expect that the throughput of whole-brain imaging will further improve. Compared with point-scanning whole-brain imaging, the BPS approach has significant advantages, with potential for imaging larger tissues, such as whole primate brains.

Recently, the CUBIC technique^18^ has been a notable breakthrough in counterstaining mouse brains. However, it remains unknown whether the chemically treated samples are sufficiently hard to facilitate mechanical sectioning, such as the samples used in STP or fMOST. Thus far, CUBIC is employed in light-sheet microscopy^19^ to image counterstained brains and has not shown potential for use in detailed whole-brain labelling and counterstaining images at axonal resolution.

Compared with current methods, BPS technology represents the technical advance of providing co-located cytoarchitecture for neural structure in the same whole brain. In addition, this technology also has the advantages of automation, high throughput, high resolution and robustness to accelerate the acquisition of high-resolution whole-brain data sets. BPS enables us to fast acquire both the neural structures and their own anatomic reference simultaneously. It helps to accurately identify and precisely annotate the brain-wide neural structures, which is difficult to be achieved by previous methods. Potentially, BPS can be routinely used to examine the intersubject variability of neurons. Consistent with previous studies^20^,^21^, the results of the present study demonstrate that in different brains of mice that are the same strain, even the same types of neurons in the same brain areas differ in terms of morphology and distribution. Revealing the intersubject variability of an axonal projection is a fundamental requirement in neuroanatomy. Axonal projections of individual neurons correlate with sublaminar location, dendritic morphology, intrinsic and synaptic properties and local connectivity patterns of those neurons, respectively^22^,^23^,^24^. The accurate tracing of axonal projections relies on the precise localization of brain regions or nuclei. Benefitting from the cytoarchitecture of the brain, the brain-wide images obtained using the BPS system can facilitate a comparison of the fine intersubject differences between axonal projections. Neuroscientists have made significant advances in the genetic labelling of specific neural circuits^25^. The combination of the genetic dissection of neural circuits and BPS technology will accelerate studies of the intersubject variability of neural circuits.

Full-volume imaging at high resolution inevitably generates large data sets, resulting in a crucial challenge to store, transfer, compute, manage and analyse these data. The most fundamental and essential challenge to mine large neural data sets is to recognize and reconstruct the complete morphology of single neurons from the raw data. This challenge is recognized worldwide as an open question and bottleneck. As a breakthrough, the BigNeuron project of the Allen Institute for Brain Science and the Blue Brain Project in Europe have focused on computational efforts. Recently, Markram *et al*.^26^ reported a simulation method of partial cortex circuits based on vast amounts of experimental and model data.

In summary, the BPS method described in the present study facilitate the acquisition of a more detailed morphology of neurons and accurately identify the brain regions or nuclei in which these are located, connected with and passing through. We propose that this method has many potential applications for neuroscience and will promote future cellular connectome studies using a traditional interval-sampling 2D atlas to precisely generate ‘personalized' 3D maps for each brain at cellular resolution. This method could also contribute to cell type, projectome and connectome studies.

## Methods

### Animals

All the animal experiments followed procedures approved by the Institutional Animal Ethics Committee of Huazhong University of Science and Technology or the Administrative Panel on Laboratory Animal Care (APLAC) at Stanford University. An 8-week-old C57BL/6J male mouse, two 8-week-old *Thy*1-GFP M-line transgenic mice (Jackson Laboratory, Bar Harbor, ME, USA), a 12-week-old *SOM*-*Cre* transgenic mouse (Huang Lab, Cold Spring Harbor Laboratory, Cold Spring Harbor, NY, USA) and a 7-week-old wild-type mouse (mixed genetic strain) were used.

### Tissue preparation

All histological procedures have been previously described^3^,^17^,^27^. Briefly, the mice were anaesthetized using a 1% solution of sodium pentobarbital and subsequently intracardially perfused with 0.01 M PBS (Sigma-Aldrich Inc., St Louis, MO, USA), followed by 4% paraformaldehyde (Sigma-Aldrich Inc., St Louis, MO, USA) and 2.5% sucrose in 0.01 M PBS. The brains were excised and post-fixed in 4% paraformaldehyde at 4 °C for 24 h. After fixation, each intact brain was rinsed overnight at 4 °C in a 0.01 M PBS solution and subsequently dehydrated in a graded ethanol series (50, 70 and 95% ethanol, changing from one concentration to the next every 1 h at 4 °C). We modified the previous resin-embedding approach^3^,^10^,^19^ to inhibit background fluorescence (Supplementary Fig. 4). Briefly, after dehydration, the brains were immersed in a graded glycol methacrylate (GMA) series (Ted Pella Inc., Redding, CA, USA), including 0.2% SBB (70%, 85%, and 100% GMA for 2 h each and 100% GMA overnight at 4 °C). Subsequently, the samples were impregnated in a prepolymerization GMA solution for 3 days at 4 °C and embedded in a vacuum oven at 48 °C for 24 h. The 100% GMA solution comprised 67 g of A solution, 2.8 g of deionized water, 29.4 g of B solution, 0.2 g of SBB and 0.6 g of AIBN as an initiator. The 70% and 85% GMA solutions (wt wt^−1^) were prepared from 95% ethanol and 100% GMA.

### Instrument

The WVT system is shown in Supplementary Fig. 1a. A mercury lamp (X-Cite exacte, Lumen Dynamics, Mississauga, Ontario, Canada) was used as the light source, providing high flexibility of wavelength selection. The collimated excitation light was directed to a digital micro-mirror device (DMD, XD-ED01N, X-digit, Shanghai, China) to generate the illumination grid pattern. The DMD comprised 1,024 × 768 electronically controllable micro-mirrors, and the pitch size of each micro-mirror was 13.68 μm. When the micro-mirror was set to ‘on', the illumination light was reflected into the light path. In contrast, in the ‘off' state, the micro-mirror blocked the illumination light. The modulated illumination light was transmitted through a lens (*f*=150 mm) and a water immersion objective (1.0 NA, XLUMPLFLN 20XW, Olympus, Shinjuku, Tokyo, Japan) and focused on the sample. The fluorescent light from the sample was collected through the objective and detected using two scientific complementary metal-oxide semiconductor cameras (ORCA-Flash 4.0, Hamamatsu Photonics K.K., Hamamatsu, Japan). The sensor array of the camera was 2,048 × 2,048 pixels with a 6.5 μm pixel size. A piezoelectric translational stage (P-725 PIFOC Long-Travel Objective Scanner, E-753 Digital Piezo Controller, PI GmbH, Karlsruhe, Germany) moved the objective for axial scanning. The actual imaging format was set to 1,700 × 1,800 pixels to fit the size of the modulated light field of the DMD. The resin-embedded whole-brain sample was subsequently fixed using cyanoacrylate in a 200 × 90 × 53 mm sample box. The sample box was screwed onto a high-precision 3D translation stage (ABL20020-ANT130-AVL125, Aerotech Inc., Pittsburgh, PA, USA). The 3D translation stage moved the sample for mosaic scanning and sectioning. The travel ranges were 200, 60 and 25 mm along the *x*, *y* and *z* axes, respectively. The encoded resolution of the *x*, *y* and *z* axes was 10, 1 and 4.5 nm, respectively. The repeatability of the *x*, *y* and *z* axes was ±200, ±75 and ±300 nm, respectively.

Sectioning and imaging were split in the WVT system, different from micro-optical sectioning tomography (MOST) and fMOST. For WVT, the microtome was used to only remove the imaged surface. The microtome was based on a 45° diamond knife (Diatome AG, Nidau, Switzerland), similar to MOST and fMOST.

We controlled the 3D stage motion for sectioning and mosaic scanning using a computer. Running data acquisition and controlling other hardware in the WVT were achieved using customized C++ acquisition software in a workstation. The acquisition and motion control software were communicated via a TCP/IP protocol. The acquired data were saved as TIFF files in a storage array (PowerVault MD1200 with a PERC H810 Host-RAID adapter, Dell Inc., Round Rock, Texas, USA).

We imaged 0.2 μm FluoSpheres Carboxylate-Modified Microspheres (Molecular Probes, Eugene, OR, USA) in the green and red channels to examine the point spread function of the WVT system. After setting the pattern period on the DMD plane to 109.44 μm (13.68 μm·pixel^−1^ × 8 pixels), a 3D image stack of the beads was acquired with a *z* step of 200 nm. We reconstructed the *x*–*z* cross-section views of a selected bead in both channels, as shown in Supplementary Fig. 1b,c. The lateral and axial full-width at half-maximum values were 0.55 and 2.20 μm in the green channel and 0.62 and 2.59 μm in the red channel, respectively (Supplementary Fig. 1d–g).

### Penetration of fluorescent nuclear staining solution

We estimated and compared the penetration performance of PI and 4′, 6-diamidino-2-phenylindole (DAPI) in the resin-embedded sample to obtain an optimized penetration protocol for the fluorescent nuclear staining solution. First, we studied the PI staining effect in GMA resin-embedded brain tissue at different concentrations and selected 2 μg ml^−1^ for use in whole-brain imaging. Then we acquired time-dependent images of the same mosaic at the depth of the imaging plane for this concentration (Supplementary Fig. 2) using the WVT. The system sectioned and imaged at a randomly selected single mosaic. The PI molecules penetrated the fresh surface immediately after sectioning. The time interval between sectioning and imaging of this mosaic was 15 s. Subsequently, we imaged this mosaic at 30 s intervals. The results indicated that the penetration of the PI solution was fast enough to facilitate staining in real time at the depth of the imaging plane. Thus, the data acquisition time was only affected through the imaging and sectioning speed and coronal plane size, not the penetration time. We also examined the features of DAPI staining and observed that the penetration of DAPI solution was too slow to achieve real-time staining. Thus, PI was used as the cytoarchitecture dye in the present study, and the staining parameters of PI were further optimized.

### Whole-brain imaging with real-time PI staining

The sample was immersed in a water bath containing PI. Whole-brain imaging was performed in the water bath. To avoid the compromising effects of sectioning marks on the machined surface, the imaging plane was set slightly below the surface, generally at 1–2 μm. Before data acquisition, we focused on the top surface of the sample and adjusted the objective to obtain a clear image. Subsequently, we moved the objective down to set the imaging plane below the surface of the sample block. After these preparations, the imaging parameters were set, and the WVT system automatically performed the sectioning and imaging to complete the brain-wide data acquisition. We also flexibly adjusted the imaging parameters, such as range of interest, exposure time and so on. To enhance the in-focus GFP signal, we added Na_2_CO_3_ into the water bath^9^. Most of the GFP molecules were preserved in a nonfluorescent state, rather than directly damaged, through chromophore protonation during the resin-embedding procedure. These fluorescent signals were chemically recovered to the fluorescent state using 0.05 M Na_2_CO_3_ during imaging.

Sectioning was achieved through a relative motion between the fixed diamond knife and the 3D translation stage in the WVT. The *x* axis of the translation stage was the sectioning direction, and the sectioning width was 2 mm. The sectioning thickness was flexible. The *y* and *z* axes provided the necessary additional movement to cover the sample surface for sectioning. Thus, a smooth fresh surface was exposed for imaging.

Subsequently, the sample was imaged. PI solution served as the immersion liquid for the objective lens. The liquid level of the solution was maintained at a level higher than the bottom surface of the objective lens during data acquisition. The GFP and PI molecules were simultaneously excited, and the emitted fluorescence signals were divided using a dichroic mirror and detected using two cameras. Three phase-shifted raw images with a phase step of *π*/2 were required to obtain an optical section image for each imaging channel^28^. The axial background inhibition was achieved through optical sectioning using SIM. When necessary, axial scanning was subsequently executed using the piezoelectric translational stage. Subsequently, the sample was moved to the next mosaic FOV with a 10 μm overlap between the adjacent FOVs. The mosaic imaging process was repeated until the entire coronal section was acquired. In addition, we used a recirculating filtration device^1^ to maintain a flattened section, remove the cutting chips, purify the PI solution and maintain a uniform PI concentration. The actual contrast of cytoarchitecture staining in the whole-brain data set showed that there was no obvious decay of the PI concentration during whole-brain imaging (Supplementary Fig. 3).

To reduce the data volume, the reconstruction of the SIM image was executed online using acquisition software. Only the reconstructed images were saved. The data set, comprising 1,700 × 1,800 pixel-sized mosaics, was saved at a 16-bit depth in the Lempel-Ziv-Welch (LZW) compression TIF format. After collection, the data set was sent to a PB-sized distributed storage via a standard 10-gigabit fibre.

We acquired the data set in Fig. 3 after sectioning at a 1 μm thickness and imaging at a 0.32 × 0.32 × 1 μm voxel size. To conserve the data acquisition time, we tried another acquisition scheme at a 0.32 × 0.32 × 2 μm voxel size for all other data acquisitions. We acquired a z-stack of two images in each FOV after axially scanning at a step of 2 μm and subsequently sectioning at a 4 μm thickness. The results (Figs 4 and 6, 7, 8) demonstrated that this acquisition scheme shortened the data acquisition time without sacrificing the axon resolution power. In addition, we also changed the mosaic scanning range according to the profile characteristics of the mouse brain to reduce useless images of the surrounding embedding medium. We achieved the rapid data acquisition of a dual-colour whole-brain data set in 77 h (Fig. 4). The data set included 561,440 mosaic images in 4,834 layers. The system performed sectioning 2,417 times and imaged 280,720 z-stacks. Sectioning lasted for a total of 24.4 s to section column by column. The imaging time of each z-stack was determined as the exposure time of the camera, phase-shift time of DMD, axial scanning time of the objective, settle time of the 3D stages and the time for online data processing. In this case, the imaging time for each z-stack was 780 ms on average. The data acquisition time was variable for different labelling technologies.

### Virus injections

AAV-CAG and AAV-CAG-FLEX expressing GFP (Serotype: 9, UNC Gene Therapy Center Vector Core, Chapel Hill, NC, USA) were used as anterograde tracers. The stereotaxic coordinates for the target areas were based on the Mouse Brain in Stereotaxic Coordinates Atlas^29^. Using a pressure injector (Nanoject II; Drummond Scientific Co., Broomall, PA, USA), 90 nl of AAV-CAG was injected into the Cg of an 8-week-old C57BL/6J mouse (0.38 mm A-P, 0.3 mm medial-lateral (M-L) and 1.8 mm dorsal-ventral (D-V)) and 90 nl of AAV-CAG-FLEX was injected into the posterior region of the anterior hypothalamic area of a *SOM*-Cre mouse (0.94 mm A-P, 0.4 mm M-L and 5 mm D-V). 0.7 μl of CAV-*Cre* and 0.5 μl AAV-FLEx(loxP)-TVA-GFP were injected into the olfactory bulb (from bregma: 0.75 mm L, 4 mm A and 1 mm V) and the ipsilateral locus coeruleus (from lambda: 0.8 mm L, 0.8 mm P and 3.2 mm V) of a 7-week-old wild-type mouse, respectively. After surgery, the animals were returned to standard living conditions for 21 days until they were sacrificed for brain sample preparation.

### Image preprocessing

Image preprocessing for mosaic stitching (Supplementary Fig. 9) and illumination correction^30^ were performed for both the GFP and PI channels. The mosaics of each coronal section were stitched to obtain an entire section based on accurate spatial orientation and neighbouring overlap. Lateral illumination correction was performed section by section. Correction coefficient along each direction was determined by calculating mean intensity along each direction and corresponding polynomial curves fitting. Equalizing the brightness of the different coronal sections was performed for axial illumination correction by quantifying the average grey-scale values of the images. Image preprocessing was implemented in C++ and optimized in parallel using the Intel MPI Library (v3.2.2.006, Intel, Santa Clara, CA, USA). Image preprocesses for mouse brain data set at the voxel resolution of 0.32 × 0.32 × 2 μm and 0.32 × 0.32 × 1 μm were executed on a computing server (72 cores, 2 GHz per core) within 6 and 12 h, respectively. All full coronal sections were saved at an 8-bit depth in LZW compression TIFF format after image preprocessing.

### Visualization and reconstruction

We visualized the data set using Amira software (v 5.2.2, FEI, Mérignac Cedex, France) to generate the figures and Movies. The preprocessed data set was imported into Amira software using a desktop graphical workstation (T7600 with two Intel E5-2687w CPUs, 256 GB memory and an Nvidia K6000 graphics card, Dell Inc., Round Rock, Texas, USA). To process the TB-sized data on a single workstation, we transformed the data format from TIFF to the native LDA type using Amira. The visualization process included extracting the data in range of interest, sampling or interpolation, reslicing the images, identifying the maximum intensity projection, volume and surface rendering, and generating the Movies using the main module of Amira. The segmentation editor module of Amira was utilized for the manual outline segmentation of whole mouse brain.

We applied the filament editor module of Amira to brain-wide tracing of long-range axons in 3D by human-machine interaction. From soma, we continuously loaded data blocks along axons and dendrites into Amira. We assigned initial and terminal points of fibres in the loaded block, and then Amira automatically calculated the path that the fibre walked along between these two points. This procedure was repeated until the whole neural morphology was reconstructed. The tracing results with original position information were saved in SWC format.

We determined the barrel columns according to the counterstained images. The original PI-labelled data were sampled to 2 × 2 × 2 μm. According to the Mouse Brain in Stereotaxic Coordinates Atlas^29^, we approximately localized the barrel cortex and resliced the sections parallel to the barrel cortex. We obtained a series of minimum intensity projections and subsequently identified the barrel columns in layer IV. According to the position information in the SWC file, we identified the corresponding coronal sections and obtained the maximum intensity projection sequences.

Besides, we used the NeuroGPS^31^ software to locate the soma in a chosen data block, as shown in Supplementary Movie 8.

Because recognition using the human eye is the golden standard of image segmenting in image processing^32^,^33^,^34^,^35^, three persons back-to-back checked all results in this manuscript to guarantee the quality of the segmented and traced neurons.

### Statistics

We compared the lengths and branch numbers of the axons, apical dendrites and basal dendrites of neurons projecting to the thalamus (THG) and midbrain (MBG). All parameters were measured using Neurolucida Explorer (MBF Bioscience, Williston, VT, USA). We performed Student's *t*-test on two groups using SPSS software (v 22, IBM, New York, USA). The confidence level was set to 0.05 (*P* value), and all results are presented as the means±s.e.m.

### Data availability

The authors declare that the data supporting the findings of this study are available within the article and its Supplementary Information files.

## Additional information

**How to cite this article:** Gong, H. *et al*. High-throughput dual-colour precision imaging for brain-wide connectome with cytoarchitectonic landmarks at the cellular level. *Nat. Commun.* 7:12142 doi: 10.1038/ncomms12142 (2016).

## Supplementary Material

Supplementary Information Supplementary Figures 1-9 and Supplementary Table 1

Supplementary Movie 1 3D reconstruction of the GFP-labeled image block shown in Fig. 3 without preprocessing.

Supplementary Movie 2 The image dataset of the whole Thy1-GFP M-line mouse brain obtained via WVT. In each section, red represents a 2-μm coronal image of PI-stained cells, and green represents a 200-μm coronal image of the maximum intensity projection of GFP-labeled neurons.

Supplementary Movie 3 3D reconstruction of a pyramidal neuron and the surrounding barrel cortex area of the Thy1-GFP M-line mouse brain. The Movie shows a pyramidal neuron and its location from layer I to the corpus callosum (corresponds to Fig. 4b). PI-stained cell bodies are in purple. The result shows that the soma of the pyramidal neuron is locating in layer V. The PI staining results clearly show layered structure of the barrel cortex and the precise locations of each cell body. The end of this Movie shows the reconstruction of the barrel walls. The size of data block is 760 × 850 × 150 μm^3^.

Supplementary Movie 4 2D consecutive slices showing the original images and axon tracing. The thickness of each slice is 16 μm, the size of display area is 220 × 160 μm, and the green signal is the tracing mark of the labeled axon on the current slice. The neuron is the green neuron in D5 in Fig. 4e.

Supplementary Movie 5 Tracing of a pyramidal neuron in the Thy1-GFP M-line mouse brain overlaid with 3D image volume rendering. The 3D image volume is rendered from 12 blocks of 320 × 320 × 300 μm^3^ that the axon passed through at the voxel size of 0.32 × 0.32 × 2 μm. The tracing of the neuron is marked in red. There is 1-μm offset between tracing mark and volume data. Tracing the same pyramidal neuron as shown in Supplementary Movie 3 overlaid with the 3D image volume rendering.

Supplementary Movie 6 Projections of pyramidal neurons in the barrel cortex of Thy1-GFP M-line brain. 3D view of Fig. 4d.

Supplementary Movie 7 3D reconstruction of the image dataset of the whole C57 mouse brain injected in Cg area with AAV-GFP and obtained via WVT. The same neuron as shown in Fig. 6. 3D volume rendering of the mouse brain is down-sampled at the voxel size of 2 × 2 × 2 μm due to the restriction of graphic card performance, and displayed with the manual outline segmentation of the whole mouse brain. Detailed volume views show the raw data in the thalamus, fibers in white and cell bodies in red. The size of the volume is 320 × 320 × 300 μm.

Supplementary Movie 8 3D volume rendering of the injection site. The same brain as shown in Supplementary Movie 7. The data size is 540 × 660 × 180 μm^3^ , and the location of the data corresponds to the block in Supplementary Figure 6a. The red spheres were detected by NeuronGPS to show the centers of each soma.

Supplementary Movie 9 2D consecutive slices showing the original images and AAV-labeled neuron tracing. The same neuron as shown in Fig. 6c.

Supplementary Movie 10 3D volume rendering of the AAV-labeled neuron. The same neuron as shown in Fig. 6c.
