# Supplementary material for: High-throughput dual-colour precision imaging for brain-wide connectome with cytoarchitectonic landmarks at the cellular level
Source: Nat Commun. 2016 Jul 4;7:12142. doi: 10.1038/ncomms12142 (PMC4932192; doi:10.1038/ncomms12142)
Supplement: Supplementary Information — Supplementary Figures 1-9 and Supplementary Table 1 [file ncomms12142-s1.pdf]

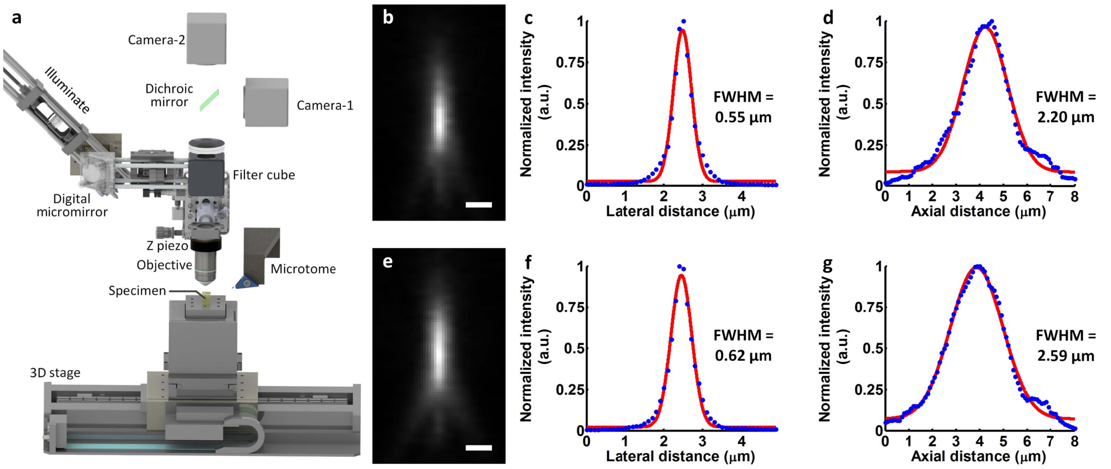


**Supplementary Figure 1 |** The WVT system. (**a**) Schematic of the system. WVT comprises a wide-field two-channel fast structured illumination microscope to accelerate imaging acquisition and a microtome to remove the imaged layer. The resin-embedded sample is fixed in a water bath (not shown) on the 3D translation stage. (**b**), (**c**) and (**d**) show x-z cross-section view, lateral and axial profiles of point spread function (PSF) in channel 1 (505/515 nm), respectively. The measured lateral and axial full width at half maximums (FWHMs) in channel 1 are approximately 0.55 and 2.20 µm, respectively. (**e**), (**f**) and (**g**) show x-z cross-section view, lateral and axial profiles of point spread function (PSF) in channel 2 (560/580 nm), respectively. The measured lateral and axial FWHMs in channel 2 are approximately 0.62 and 2.59 µm, respectively.

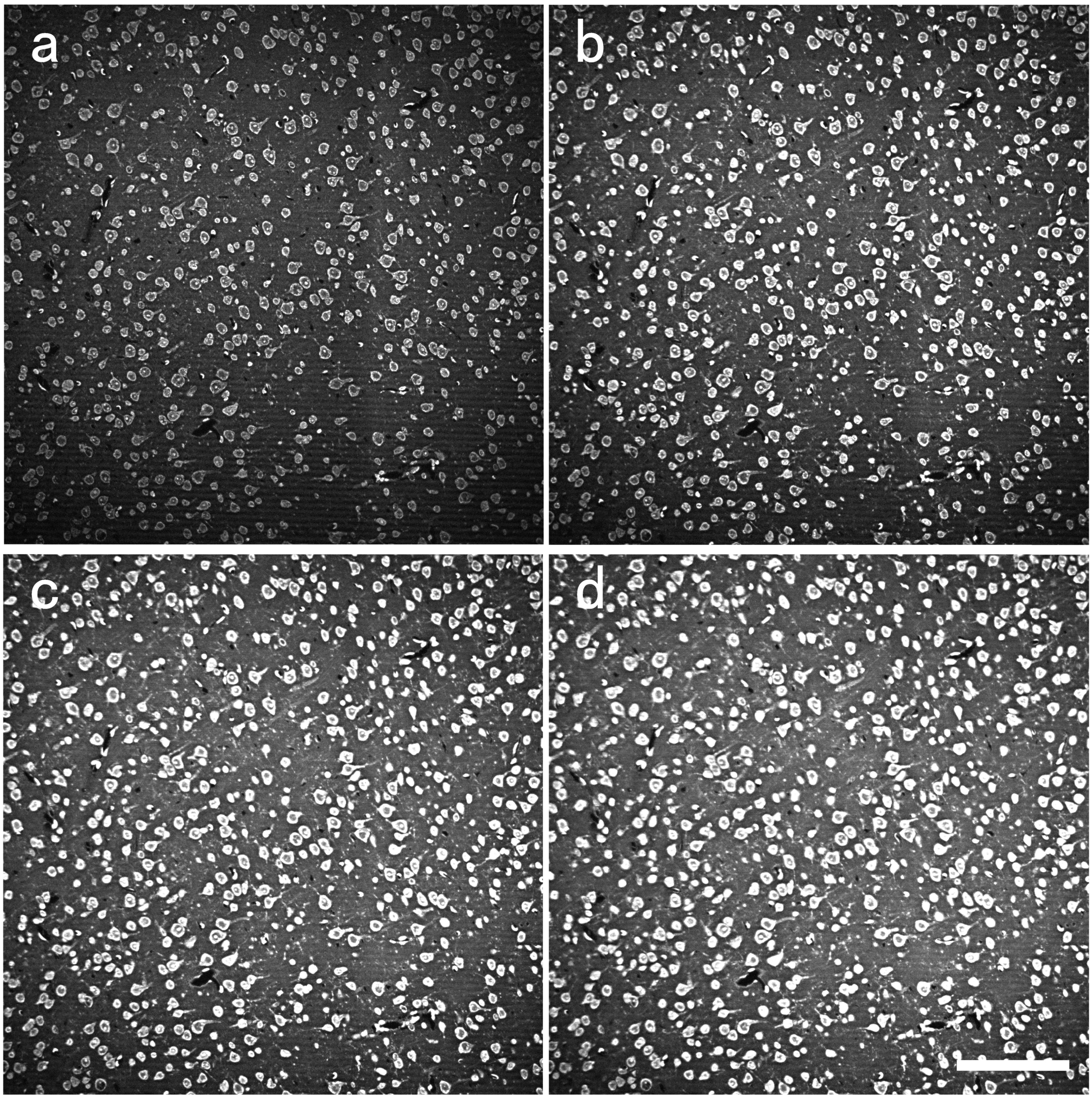


**Supplementary Figure 2 |** Penetration of PI staining in GMA resin-embedded brain tissue. **a-d**: Images at penetration times of 15, 45, 75, and 105 s obtained through WVT. The exposure time was 9 ms. Scale bar: 100 μm.

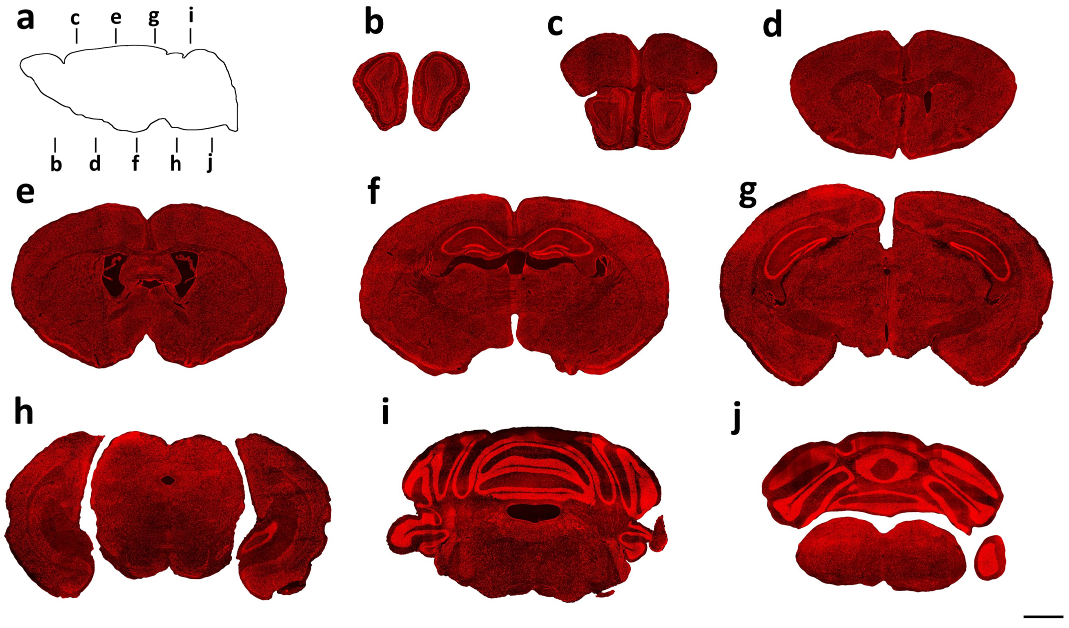


**Supplementary Figure 3 |** PI-stained images of the Thy-1 GFP mouse brain obtained from the same dataset shown in Fig. 4. (**a**) Locations of the images shown in (**b**) to (**j**), spaced 1.0 mm apart, from the olfactory bulb to the cerebellum. (**b** to **j**) A series of coronal plane images. The scale bar is 1 mm.

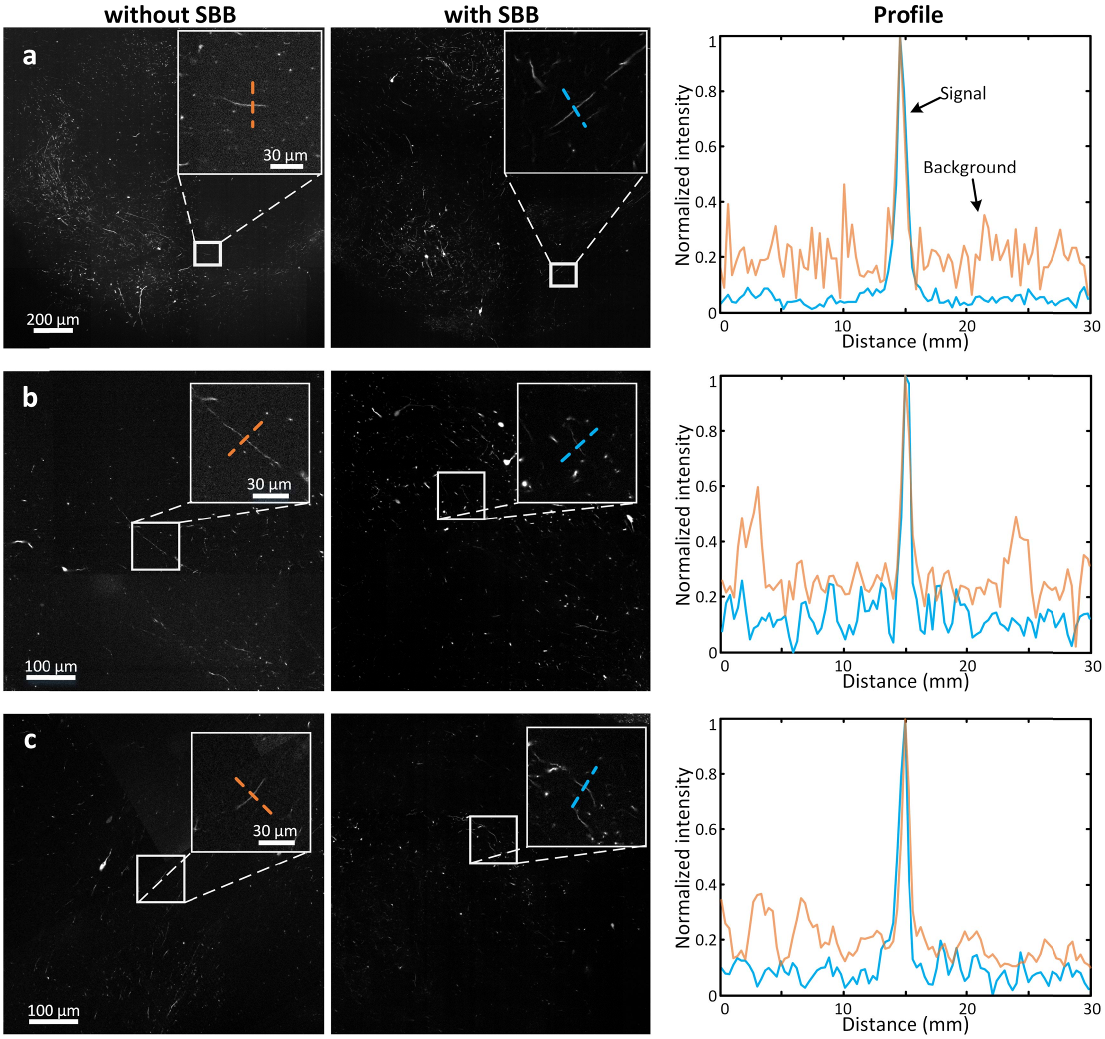


**Supplementary Figure 4 |** SNR improvement after SBB treatment. Images and SNR comparison of the three groups of non-treated (left column) and SBB-treated (middle column) Thy1-eGFP-M mouse brain tissue samples embedded in GMA. The images were obtained through WVT. The orange and blue lines in the charts (right column) show the intensity profiles (measured using ImageJ on 8-bit TIF images) along the orange and blue dashed lines in **a-c**, respectively. The images of the SBB-treated samples show SNR improvement compared with the non-treated samples.

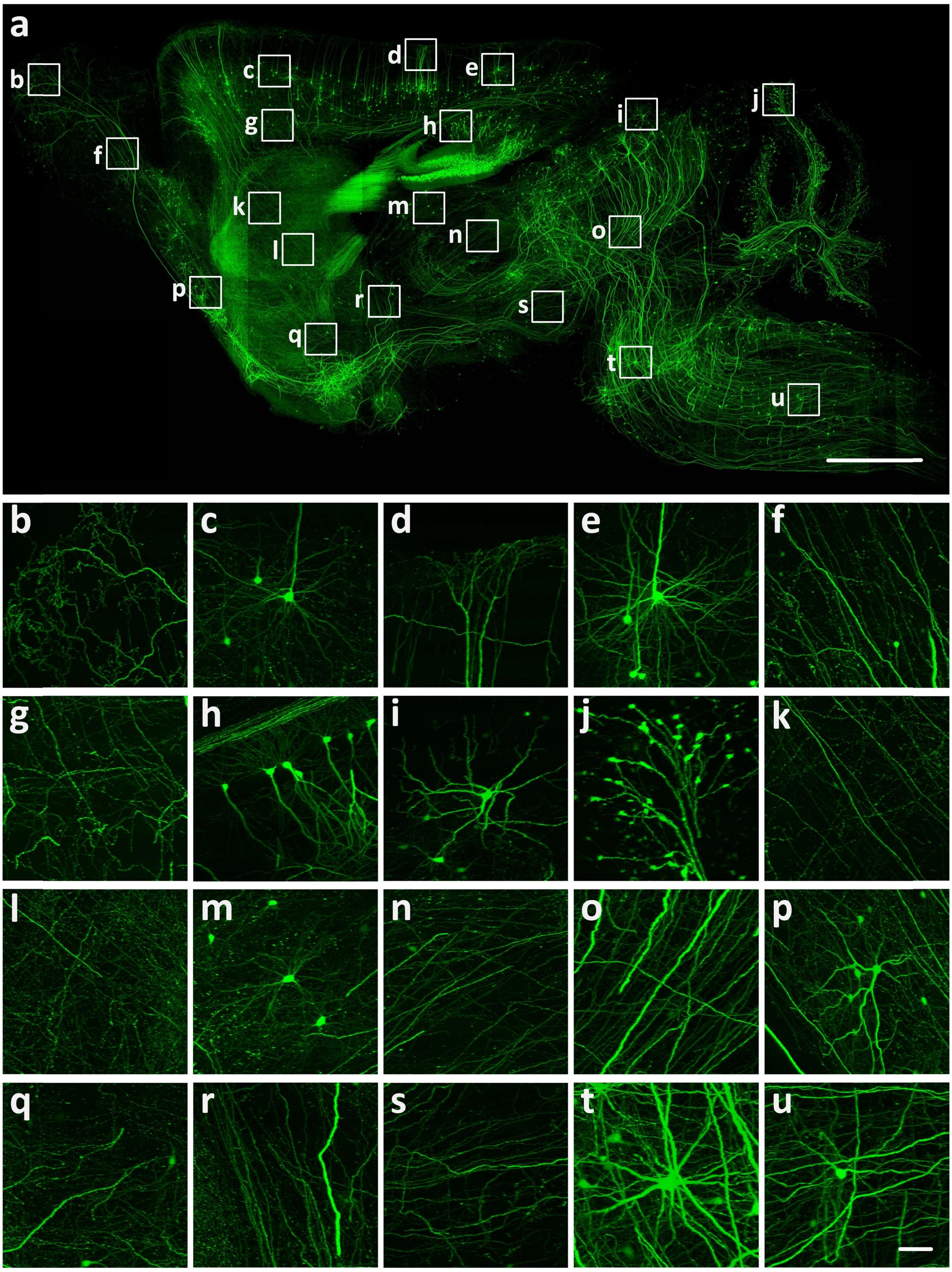


**Supplementary Figure 5 |** Sagittal reconstruction of the maximum intensity projections in the Thy-1 GFP M-line mouse brain. (b)-(u) Enlarged views of white rectangles shown in (a), respectively. The raw data obtained from the same dataset shown in Fig. 2. The total projection thickness shown in (a) and other subimages are 500 µm and 250 µm, respectively. The sizes of (b)-(u) are 300 × 300 $\mu m^2$. The scale bar in (a) and other subimages are 1 mm and 50 µm, respectively.

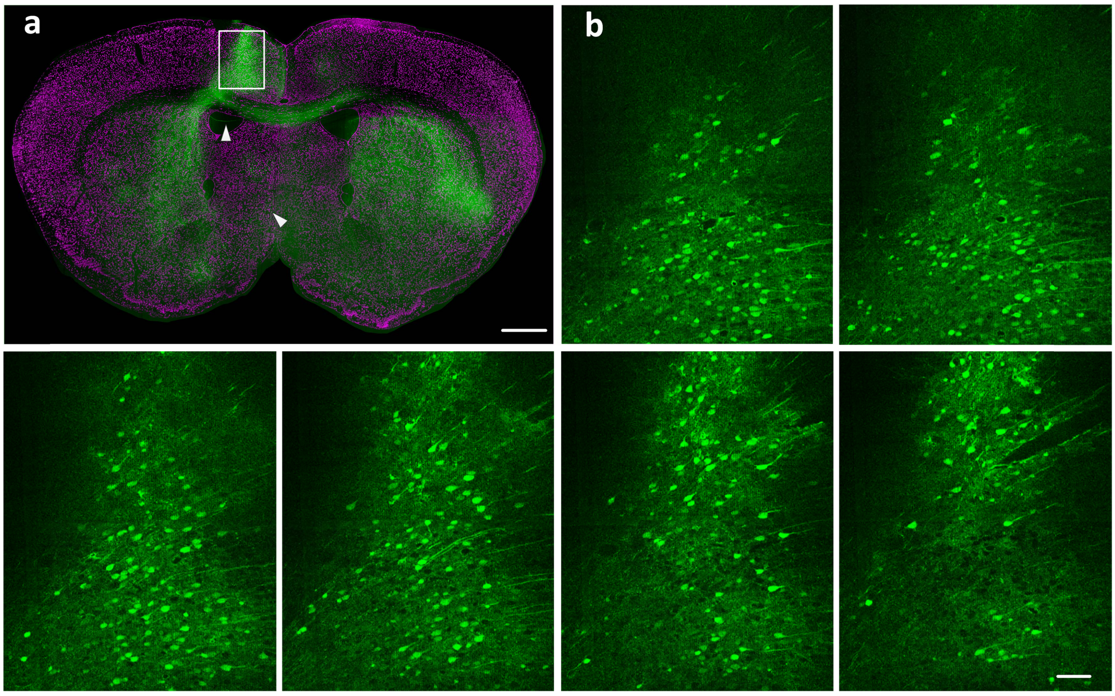


**Supplementary Figure 6 |** Single-neuron soma discrimination in dense coronal images around the injection site. (**a**) Maximum intensity projections of the coronal section around the injection site in the Cg cortex in the C57 mouse (the same sample as shown in **Figure 6c**). (**b**) Six serial raw images of the GFP signal around the injection sites shown in the white box of (**a**). The thickness of each raw image is 2 μm. A single neuron soma could be distinguished in these continuous sections, although the neuron appears dense in (**a**). This figure corresponds to **Supplementary Movie 8**. Scale bar: (a) 500 μm and (b) 50 μm.

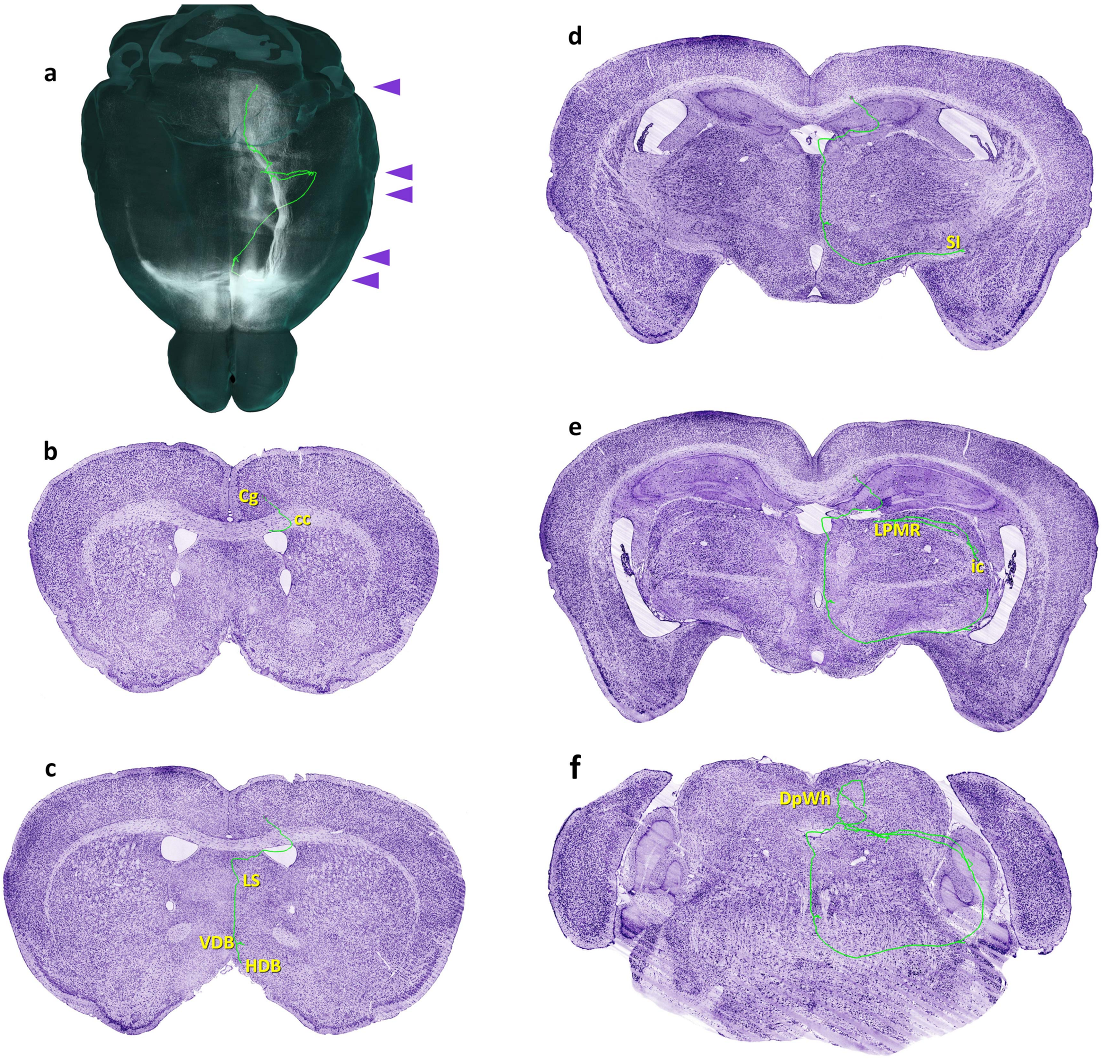


**Supplementary Figure 7 |** Continual single-axon tracing in a C57 mouse brain labeled with AAV-GFP. (**a**) 3D reconstruction of the whole mouse brain along the horizontal direction. Anterograde projections from the Cg primarily distributed in the ipsilateral hemisphere. A single axon is reconstructed and shown in the green line (same as shown in **Fig. 6d**). (**b**-**f**) Manually tracing the single neuron along the A-P direction according to PI-stained images (purple). The coronal sections are indicated by purple arrowheads along the A-P direction in (**a**). The thickness of the section is 2 µm. This figure corresponds to **Supplementary Movies 9 and 10**.

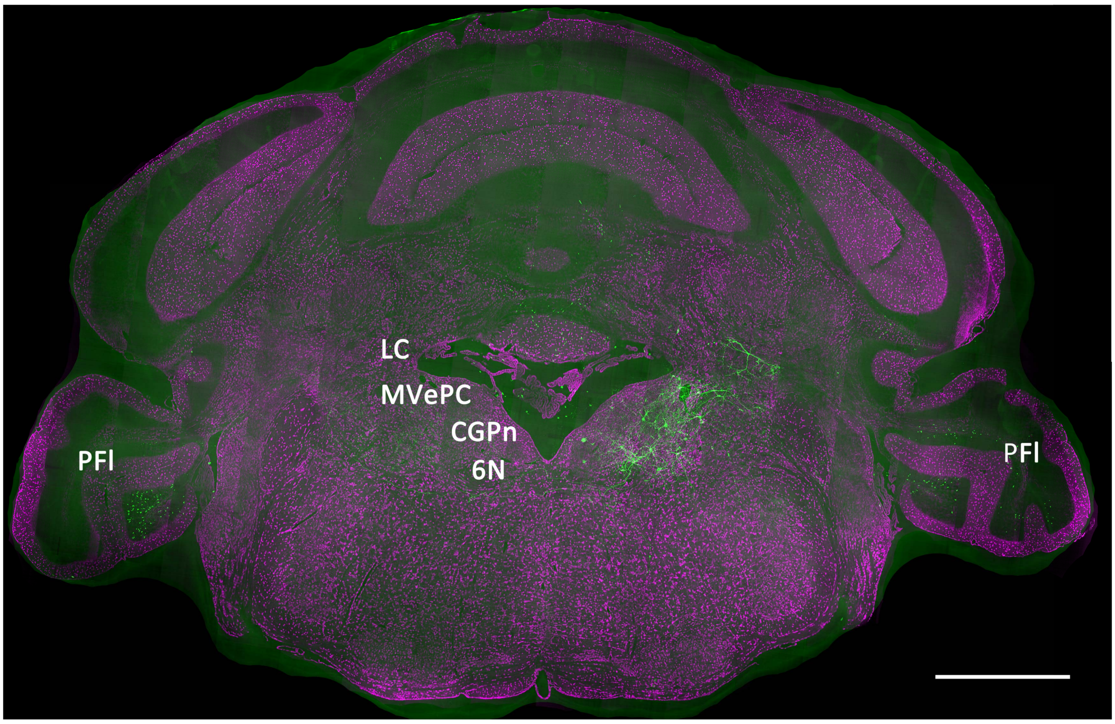


**Supplementary Figure 8 |** Visualization of sparse labeling neurons using cytoarchitecture. The green channel showed the same maximum intensity projections in the coronal sections as indicated in **Fig. 8(a)**. Magenta represents PI-stained cells (2 μm thickness). Abbreviation: (6N) abducens nucleus, (CGPn) central gray of the pons, (LC) locus coeruleus, (MvePC) medial vestibular nucleus, parvicellular part, (PFl) paraflocculus. The scale bar is 1 mm.

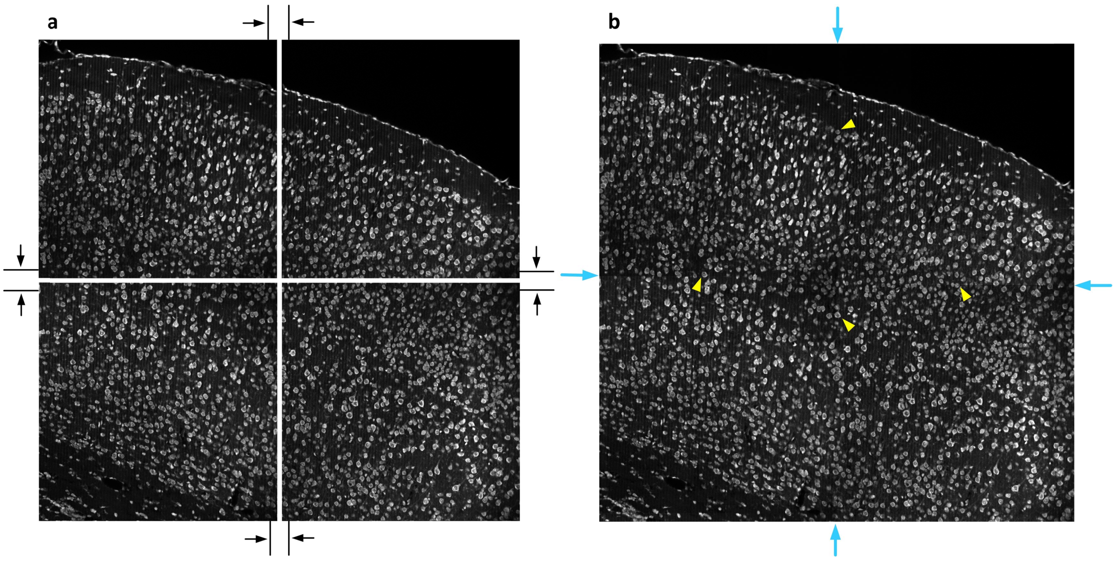


**Supplementary Figure 9 |** Registration of neighboring mosaics on the same coronal plane. (**a**) Raw PI-stained section from the cortex after stitching 2 × 2 mosaics. The black lines indicate overlapping between the neighboring mosaics, and the black arrows represent the stitching directions. (**b**) Stitched image. The blue arrows indicate the locations of the seams of the neighboring mosaics. The yellow arrowheads indicate the entire shape of the PI-stained soma at the seams, confirming the integrity and continuity of the dataset.

| Neuron No.* | Dataset ID | Barrel | Longest projection region | Other projection regions | Axon length(μm) | Total number of axon branch | Dendrite length(μm) | Total number of dendrite branch | Apical dendrite length(μm) | Total number of apical dendrite branch | Basal dendrite length(μm) | Total number of basal dendrite branch |
|---|---|---|---|---|---|---|---|---|---|---|---|---|
| 1 | Dataset1 | A1 | CP | | 1623.0 | 3 | 3211.1 | 58 | 1701.5 | 32 | 1509.6 | 26 |
| 2 | Dataset1 | A4 | TH | CP | 2821.9 | 12 | 887.7 | 9 | 642.9 | 3 | 244.8 | 6 |
| 3 | Dataset1 | B2 | TH | | 2975.2 | 5 | 1914.4 | 47 | 1321.1 | 34 | 593.3 | 13 |
| 4 | Dataset2 | B2 | TH | | 4668.0 | 11 | 1921.1 | 32 | 1042.7 | 17 | 878.4 | 15 |
| 5 | Dataset1 | B3 | MB | | 4030.7 | 1 | 2365.4 | 40 | 1395.2 | 25 | 970.2 | 15 |
| 6 | Dataset1 | B3 | MB | TH | 6265.2 | 5 | 6645.3 | 98 | 5984.9 | 89 | 660.4 | 9 |
| 7 | Dataset1 | B3 | TH | ipsilateral SSs | 2987.6 | 6 | 1951.6 | 47 | 953.3 | 20 | 998.3 | 27 |
| 8 | Dataset1 | C2 | MB | | 4836.2 | 1 | 3483.8 | 64 | 2506.5 | 49 | 977.3 | 15 |
| 9 | Dataset2 | C2 | MB | | 7910.7 | 5 | 8248.4 | 95 | 4126.6 | 47 | 4121.8 | 48 |
| 10 | Dataset1 | C4 | TH | | 2722.7 | 1 | 3228.7 | 76 | 1914.8 | 46 | 1313.9 | 30 |
| 11 | Dataset2 | C4 | TH | | 2524.6 | 1 | 2126.6 | 36 | 1230.3 | 18 | 896.3 | 18 |
| 12 | Dataset1 | C5 | CP | | 1083.2 | 1 | 1607.8 | 34 | 1326.8 | 27 | 281.0 | 7 |
| 13 | Dataset2 | C5 | MB | | 10303.6 | 3 | 7522.6 | 114 | 5208.3 | 74 | 2314.3 | 40 |
| 14 | Dataset2 | C5 | TH | | 2787.9 | 5 | 2003.8 | 49 | 1202.0 | 24 | 801.8 | 25 |
| 15 | Dataset1 | D1 | MB | cst | 6125.0 | 9 | 3498.9 | 65 | 2837.3 | 53 | 661.6 | 12 |
| 16 | Dataset1 | D1 | TH | | 3070.4 | 1 | 3152.6 | 61 | 1962.0 | 40 | 1190.6 | 21 |
| 17 | Dataset1 | D2 | MB | cst | 8174.9 | 7 | 4265.3 | 58 | 2440.6 | 33 | 1824.7 | 25 |
| 18 | Dataset2 | D2 | MB | | 3798.7 | 1 | 3986.8 | 70 | 2335.3 | 39 | 1651.5 | 31 |
| 19 | Dataset2 | D2 | MB | cst | 6080.3 | 5 | 7381.1 | 109 | 3531.5 | 51 | 3849.6 | 58 |
| 20 | Dataset1 | D5 | MB | TH, cst | 6571.9 | 9 | 7299.1 | 119 | 5143.9 | 85 | 2155.2 | 34 |
| 21 | Dataset1 | D5 | CP | | 856.8 | 1 | 1198.3 | 27 | 790.7 | 18 | 407.6 | 9 |
| 22 | Dataset1 | D5 | TH | | 4421.7 | 7 | 1304.8 | 33 | 922.9 | 24 | 381.9 | 9 |
| 23 | Dataset2 | D5 | TH | | 7234.8 | 15 | 2766.8 | 49 | 1653.9 | 33 | 1112.9 | 16 |
| 24 | Dataset1 | E2 | TH | | 4662.4 | 7 | 3509.3 | 50 | 1795.9 | 27 | 1713.4 | 23 |
| 25 | Dataset2 | E4 | cc | | 1169.2 | 1 | 2159.1 | 34 | 1001.6 | 15 | 1157.5 | 19 |

| | | | | | | | | | | | | |
|---|---|---|---|---|---|---|---|---|---|---|---|---|
| 26 | Dataset1 | E5 | CP | | 657.5 | 1 | 1498.4 | 39 | 796.8 | 21 | 701.6 | 18 |
| 27 | Dataset1 | E5 | TH | | 3346.1 | 11 | 2400.6 | 43 | 1317.1 | 24 | 1083.5 | 19 |
| 28 | Dataset1 | septa | CP | | 2872.8 | 1 | 5487.3 | 106 | 2957.8 | 61 | 2529.5 | 45 |
| 29 | Dataset1 | septa | TH | | 2307.4 | 3 | 929.0 | 18 | 582.7 | 8 | 346.3 | 10 |
| 30 | Dataset1 | septa | TH | | 3188.9 | 3 | 1925.5 | 38 | 1140.9 | 26 | 784.6 | 12 |
| 31 | Dataset1 | septa | TH | | 2769.2 | 3 | 3385.1 | 57 | 2281.0 | 35 | 1104.1 | 22 |
| 32 | Dataset2 | septa | MB | cst | 12514.8 | 18 | 9729.8 | 145 | 6031.3 | 89 | 3698.5 | 56 |

**Supplementary Table 1 |** Morphology of long-range neurons of THG and MBG

Abbreviations: cc=corpus callosum, cst=corticospinal tract, CP=Caudoputamen, MB=Midbrain, SSs=Supplemental somatosensory area, TH=Thalamus.

* Blue and green represent neurons with axons extending the furthest to the striatum or thalamus and the midbrain, respectively.
